# Assessment of radial artery atherosclerosis in acute coronary syndrome patients: an in vivo study using optical coherence tomography

**DOI:** 10.1186/s12872-022-02561-5

**Published:** 2022-03-21

**Authors:** Zixuan Li, Zhe Tang, Yujie Wang, Zijing Liu, Guozhong Wang, Libin Zhang, Yongxia Wu, Jincheng Guo

**Affiliations:** 1 Department of Cardiology, Beijing Luhe Hospital, Capital Medical University, Beijing, China; 2 Department of Cardiology, Beijing Anzhen Hospital, Capital Medical University, Beijing, China

**Keywords:** Optical coherence tomography, Atherosclerosis, Radial artery, Peripheral artery disease

## Abstract

**Background:**

Radial artery (RA) atherosclerosis in acute coronary syndrome (ACS) patients has not been systematically observed in vivo. The study aims to characterize plaque morphology and intimal hyperplasia of the RA in patients with ACS, using optical coherence tomography (OCT).

**Methods:**

In this retrospective study involving 239 ACS patients underwent RA OCT without guidewire shadow, 3 groups were divided according to the following criteria: radial artery plaque (RAP) group included patients with fibrous, lipid or calcified plaque; patients without RAP were further classified into radial intimal hyperplasia (RIH) group (intima media thickness ratio [IMR] ≥ 1) or normal group (IMR < 1). The presence and characteristics of RAP and its related risk factors were identified.

**Results:**

The RAP, RIH and normal groups included 76 (31.8%), 69 (28.9%) and 94 (39.3%) patients, respectively. Patients in RAP group were the oldest, compared with those in the RIH and normal groups (*p* < 0.001), and more frequently had triple vessel disease (*p* = 0.004). The percentage of plaque rupture (72.4% vs. 56.4%, *p* = 0.018) and calcification (42.1% vs. 27.6%, *p* = 0.026) at culprit lesion were significantly higher in patients with RAP than those without RAP. A total of 148 RAP were revealed by OCT, including fibrous (72, 48.6%), lipid (50, 33.8%) and calcified plaques (26, 17.6%). The microvessels were also frequently observed in the RAP group than that in RIH and normal groups (59.2% vs. 8.7% vs. 9.6%, *p* < 0.001). Multivariate logistic regression analysis showed that age, diabetes, and smoking history (all *p* < 0.05) were independent risk factors for RAP.

**Conclusions:**

In terms of insights gained from OCT, RA atherosclerosis is not uncommon in ACS patients by OCT, sharing several morphological characters with early coronary atherosclerosis. Aging, diabetes, and smoking are risk factors for RAP.

**Supplementary Information:**

The online version contains supplementary material available at 10.1186/s12872-022-02561-5.

## Background

Atherosclerosis is a systemic disease that may involve multiple vessels such as the carotid, coronary, and peripheral arteries. As a manifestation of systemic atherosclerosis, radial artery (RA) atherosclerosis has received less attention than other atherosclerotic diseases, leading to under-diagnosis [1]. Previous studies using high-resolution ultrasound have demonstrated that the RA represents a potentially useful window for in vivo evaluation of systemic atherosclerosis [2–5]. Limited evidence from small histopathological and ultrasound studies has shown that intimal hyperplasia and atherosclerosis burden in the RA is associated with several traditional cardiovascular risk factors and confer diagnostic and prognostic information on coronary artery disease (CAD) [2, 3, 6–10]. However, the presence of atherosclerosis and plaque components in the RA of acute coronary syndrome (ACS) patients might be underestimated because of limited diagnostic modalities. Intraluminal optical coherence tomography (OCT) provides real-time visualization of arterial structures and precise measurements at near histological resolution, and is commonly used for coronary artery observation [11, 12].

The present study employed OCT examination without guide wire shadow to characterize plaque morphology and the extent of intimal hyperplasia of the RA in patients with ACS, and explored the association of RA atherosclerosis with risk factors.

## Methods

### Study design and population

This was a retrospective, single-center study conducted by reviewing the OCT database of the Beijing Luhe Hospital between March 2019 and September 2020. A total of 239 patients with ACS who underwent RA OCT examination at the end of transradial OCT-guided coronary intervention were enrolled. Exclusion criteria were previous history of ipsilateral RA cannulation, abnormal Allen test, poor image quality such as incomplete blood washout, or data missing. The study flow chart is shown in Additional file 1: Figure S1. The study was approved by the ethics committees of Beijing Luhe Hospital, Capital Medical University and is in accordance with the declaration of Helsinki. Informed consent was obtained from all subjects.

Patients with the occurrence of at least one visible radial artery plaque (RAP) were classified as RAP group. Patients without RAP were further divided into two subgroups according to intima-media thickness ratio (IMR) [13, 14]: patients with intimal hyperplasia (denoted the radial intimal hyperplasia [RIH] group, IMR ≥ 1) and those with normal RA (normal group, IMR < 1). Baseline clinical parameters were compared among the normal, RIH, and RAP groups.

### OCT image acquisition

All procedures were performed by two experienced high-volume interventional cardiologists. After successful coronary procedure, RA angiography was used to define the location of radioulnar bifurcation, by administering a cocktail consisting of 2.5 mg verapamil and 0.2 mg nitroglycerin intra-arterially through the sheath. Then the RA sheath (6F, external diameter 2.48 mm, Terumo Co, Tokyo, Japan) was retracted from the RA, leaving 2 cm inside the RA. An X-ray contrast ruler was used to determine the correct starting position of OCT to a distance 150 mm proximal to the actual sheath tip. For imaging, the OCT catheter was advanced via angioplasty wire; once in position, the angioplasty wire was withdrawn. Three OCT pullback recordings (20 mm/s) including proximal (0‒50 mm), middle (50‒100 mm), and distal (100‒150 mm) portions were performed using saline to flush the RA (Additional file 1: Figure S2A). The OCT procedure was performed using a C7XR FD-OCT system (St. Paul, MN, USA).

### Definition and measurement

The OCT images were digitally stored and every single frame (0.2 mm) analyzed offline by two experienced doctors, who were blinded to the clinical data, using proprietary software (LightLab Imaging) with confirmation of the correct calibration settings for Z-offset.

**Fig. 1:**
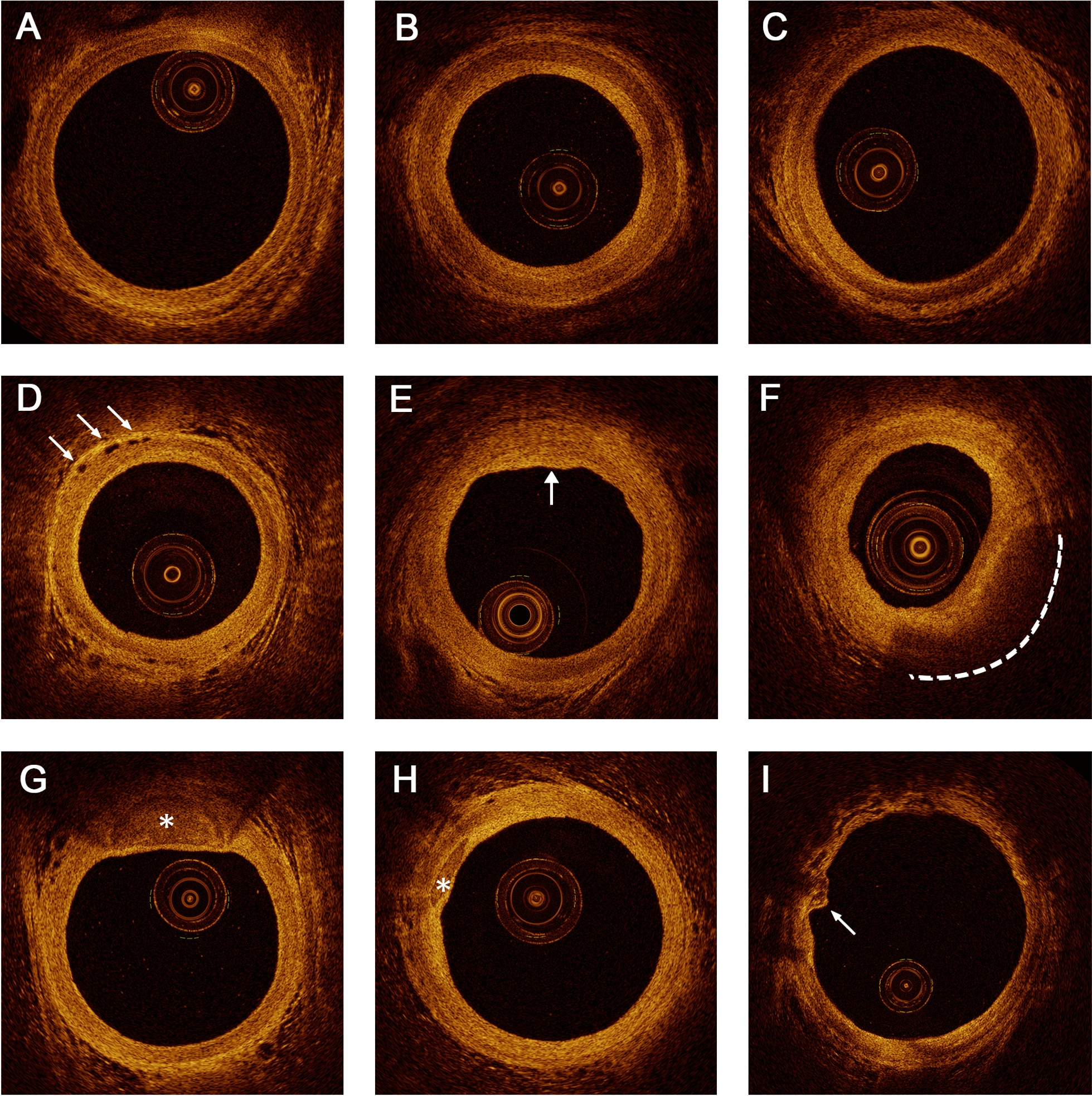
Representative optical coherence tomography images. **A** Normal RA with visible three-layer structure. **B** Concentric intimal hyperplasia in RA: the intima was of uniform thickness, being thinner or only as thick as than the media. **C** RA with eccentric intimal hyperplasia at 7 to 9 o’ clock positions. **D** microvessels (arrowheads). **E** Fibrous plaque (arrowhead) without lipid or calcium component. A signal-rich, homogeneous region is typical for fibrous tissue. **F** Lipid pool (dotted line**)** covered by thick fibrous cap. **G**–**H** Calcified plaque (asterisk). **I** Calcified protrusion (arrowhead). RA, radial artery

The OCT image interpretation was according to the consensus guidelines [15–17]. The intimal hyperplasia of the signal-rich layer nearest the lumen was defined by IMR [7, 11, 14]. RAP was identified by OCT as segments with a loss of the normal 3-layered structure of the vessel wall [11, 15]. The plaque type was classified as: (1) fibrous plaque (high backscattering and homogeneous signal-rich regions); (2) lipid plaque (heterogenic, signal-poor, highly attenuating intimal regions with diffuse or poorly defined borders); and (3) calcified plaque (signal-poor or heterogeneous region with a sharply delineated border). Additionally, microvessels were characterized by a black hole that was sharply delineated for at least three consecutive frames (diameter of 50–300 µm) (Fig. 1). The number of atherosclerotic plaques and longitudinal length of plaque involvement were recorded (Additional file 1: Figure S3); two plaques were seen as separated when their distance was more than 10 mm on the longitudinal view. A diffuse lesion was defined as longer than 20 mm. The maximal lipid or calcified arc and the fibrous cap thickness were recorded. The maximal depth of calcified plaque was also determined. A detailed description of coronary atherosclerotic plaques by OCT is included in the Additional file 1.

The structural parameters included lumen diameter and area, the internal and external elastic laminae area, and intimal and medial thickness. Other indices of the extent of intimal hyperplasia included (1) percentage of luminal narrowing = (intimal area + medial area)/external elastic lamina area × 100; (2) intimal thickness index (ITI) = intimal area/medial area; (3) IMR = maximal intimal thickness (MIT)/width of media at maximal intimal thickness; and (4) intimal eccentricity index (IEI) = MIT/minimum intimal thickness [6, 7, 13, 14] (Additional file 1: Figure S2B). Measurements were only recorded in the cross-sectional frames free of atherosclerotic plaque, and near the designated site was considered a viable alternative if artifacts or side branches obscured a significant portion (> 90°). Measurements were repeated three times and the mean values take for the final calculation.

### Reproducibility of OCT RAP assessment

To assess interobserver variability, an author (TZ) who was blinded to clinical, angiographical, and OCT data selected 50 cases, for which all annotations on the OCT images were deleted. Two experienced OCT observers (GJC and TZ) then interpreted the 50 OCT cases, and an OCT observer (GJC) assessed them again at 4-week intervals. The interobserver Kappa coefficients were 0.975 for the reproducibility of plaque occurrence, 0.919 for fibrous plaque, 0.751 for lipid plaque, and 0.963 for calcified plaque. Any inconsistent results between the two observers were settled by consensus.

### Statistical analysis

Categorical variables were presented as numbers and percentages and were compared using the Chi-square tests or Fisher’s exact test, as appropriate. Continuous variables were expressed as mean ± standard deviation (SD) for normally distributed variables and as median [interquartile ranges (IQR)] for non-normally distributed variables. The Kolmogorov–Smirnov test was performed to assess data normality. For comparisons, two-sided Student’s t-test, Mann–Whitney U test, or Kruskal–Wallis test were used as appropriate, and analysis of variance was conducted among the three groups (normal, RIH, and RAP groups) followed by post-hoc test, with an overall significance level of 0.05. A Bonferroni correction was used to control for multiple comparisons. Spearman correlation coefficient was used for the determination of correlations. Multivariate logistic regression analysis was conducted to determine RAP predictors. The statistical analyses were performed using statistical software (SPSS version 23, Chicago, IL, USA).

## Results

### Baseline characteristics

The baseline characteristics of the 239 ACS patients including RAP, RIH and normal groups are summarized in Table 1. Compared with those in the RIH and normal groups, patients in the RAP group were the oldest (*p* < 0.001), and had a higher prevalence of diabetes mellitus, hypertension, and history of stroke. Patients in the RAP group more commonly had myocardial infarction, followed by the RIH and normal groups (94.7% vs. 92.8% vs. 81.9%, *p* = 0.032). Patients in the RAP group also had more triple vessel disease (TVD) than those in the RIH and normal groups (43.4% [RAP] vs*.* 24.6% [RIH], p = 0.017; 43.4% [RAP] vs. 21.3% [normal], *p* = 0.002). Other variables, including hyperlipidemia, family history of CAD, prior myocardial infarction, and laboratory variables were comparable among the groups.

**Table 1:** Patient CHARACTERISTICS (N = 239)

Variable	Normal group (n = 94)	RIH group (n = 69)	RAP group (n = 76)	*P*
Age (years)	47.2 ± 9.6	61.1 ± 10.4^a^	64.2 ± 10.0	< 0.001
Males	88 (93.6)^a,b^	46 (66.7)	56 (73.7)	< 0.001
Body mass index (kg/m^2^)	26.5 ± 3.3	26.3 ± 3.3	25.9 ± 3.3	0.472
Current or previous smoking	71 (45.5)^a^	33 (21.2)	52 (33.3)^c^	0.001
Diabetes mellitus	17 (18.1)	19 (27.5)	27 (35.5)^b^	0.036
Hypertension	39 (41.5)	40 (58.0)^a^	47 (61.8)^b^	0.017
Hyperlipidemia	72 (76.6)	47 (68.1)	61 (80.3)	0.215
Previous stroke	1 (1.1)	5 (7.2)	10 (13.2)^b^	0.007
Previous MI	1 (1.1)	0	1 (1.3)	–
Peripheral vascular disease	3 (3.2)	2 (2.9)	3 (3.9)	1.000
Renal insufficiency	2 (2.1)	2 (2.1)	8 (10.5)	0.051
Family history of CAD	17 (18.1)	9 (13.0)	8 (10.5)	0.359
Clinical presentation				
MI	77 (81.9)	62 (89.9)	72 (94.7)^c^	0.032
Unstable angina	17 (18.1)	7 (10.1)	4 (5.3)	
Triple vessel disease	20 (21.3)	17 (24.6)	33 (43.3)^b,c^	0.004
Multivessel disease	49 (52.1)	44 (63.8)	57 (75.0)^b^	0.009
Procedure characteristics				
CAG	15 (16.0)	6 (8.7)	4 (5.3)	0.064
PCI	93 (84.0)	64 (91.3)	57 (94.7)	
Medications before admission				
Antiplatelet	1 (1.1)	4 (5.8)	7 (9.2)^b^	0.037
Statin	4 (4.3)	3 (4.3)	7 (9.2)	0.394
CCB	20 (21.3)	13 (18.8)	17 (22.4)	0.901
Beta blocker	9 (9.6)	2 (2.9)	5 (6.6)	0.248
ACEI or ARB	12 (12.8)	14 (20.3)	18 (15.8)	0.425
Biochemistry data				
LDL cholesterol (mg/dl)	122.8 ± 34.9	115.4 ± 34.4	114.5 ± 35.1	0.233
Serum creatinine (μmmol/L)	77.1 ± 17.6	75.5 ± 14.2	76.9 ± 16.4	0.808
Procedure time, min	77.1 ± 28.6	80.4 ± 31.7	71.9 ± 32.4	0.285
Contrast media volume, ml	205 ± 67	203 ± 61	194 ± 76	0.581

*ACE* angiotensin-converting enzyme, *ARB* angiotensin II receptor blocker, *CAD* coronary artery disease, *CAG* coronary angiogram, *CCB* Calcium channel blocker, *LDL* low-density lipoprotein, *MI* myocardial infarction *PCI* percutaneous coronary intervention, *RIH* radial artery intimal hyperplasia, *RAP* radial artery plaque*, SD* standard deviation

Renal insufficiency: estimated glomerular filtration < 60 ml/min/1.73 m^2^

Values are mean ± SD, n (%), or median (25th, 75th percentiles)

*p* < 0.05 for ^a^normal versus RIH; ^b^normal versus RAP; ^c^RIH versus RAP

### Incidence and characteristics of RAP

#### Patient level

Among the 76 (31.8%) subjects presenting with RAP, 67.1% had multiple segment involvement and 32.9% had single segment involvement. More patients presented with RAP in the distal and proximal segments than in the middle segment (20.9% vs. 19.7% vs. 11.7%, *p* = 0.016), especially for lipid plaque (*p* = 0.008). The microvessels were frequently observed in the RAP group than that in RIH and normal groups (59.2% vs. 8.7% vs. 9.6%, *p* < 0.001), which were more common present in the distal (20.5%) segment, followed by the mid (12.6%) and proximal (6.7%) segments (*p* < 0.001). (Table 2 and Additional file 1: Table S1).

**Table 2:** Longitudinal distribution of atherosclerotic plaque in RA

Variables	All	Segments			*P*^a^
		Proximal 0–50 mm	Middle 50–100 mm	Distal 100–150 mm	
*Patient-level*					
Plaque present	76 (31.8)	47 (19.7)^b^	28 (11.7)	50 (20.9)^c^	0.016
Fibrous	50 (20.9)	28 (11.7)	18 (7.5)	24 (10.0)	0.300
Lipid	39 (16.3)	20 (8.4)^b^	6 (2.5)	21 (8.8)^c^	0.008
Calcified	15 (6.3)	5 (2.1)	6 (2.5)	14 (5.9)^d^	0.049
Total lesion length, mm	7.1 (3.2–13.1)	4.9 (2.5–9.5)	5.4 (2.1–9.9)	6.0 (3.1–13.5)	0.394
Microvessel present	60 (25.1)	16 (6.7)	30 (12.6)^b^	49 (20.5)^c,d^	< 0.001
Macrophage present	29 (12.1)	7 (2.9)	10 (4.2)	19 (7.9)^d^	0.033
*Plaque-level*					
Number of Plaques	148	55 (37.2)	30 (20.3)	63 (42.6)	
Plaque phenotype					
Fibrous	72 (48.6)	29 (40.3)	18 (25.0)	25 (34.7)	
Lipid	50 (33.8)	21 (42.0)	6 (12.0)	23 (46.0)	
Calcified	26 (17.6)	5 (19.2)	6 (23.1)	15 (57.7)	
Quantitative analysis					
Plaque (mm)	4.9 (2.3–8.3)	3.6 (2.1–7.8)	5.5 (2.1–7.8)	4.8 (3.0–8.4)	0.389
Fibrous (mm)	3.4 (1.6–7.7)	3.3 (1.3–8.1)	5.0 (1.7–7.4)	3.1 (1.6–8.7)	0.841
Calcified (mm)	8.1 (4.6–20.8)	10.3 (2.0–19.1)	14.2 (4.3–29.5)	7.8 (4.7–16.3)	0.806
Maximal depth (mm)	0.21 (0.16–0.33)	0.16 (0.12–0.22)	0.17 (0.14–0.23)	0.27 (0.18–0.56)	0.049
Maximum arc (°)	62.8 (43.2–101.7)	55.9 (19.3–82.3)	70.2 (44.9–119.6)	66.2 (43.8–101.0)	0.470
FCT (μm)	120 (40–170)	60 (40–170)	90 (30–120)	130 (40–250)	0.389
Lipid (mm)	4.9 (3.1–6.9)	4.9 (2.6–6.7)	5.4 (2.5–9.0)	4.8 (3.3–7.0)	0.812
FCT (μm)	220 (140–330)	180 (120–280)	420 (210–640)	250 (140–340)	0.091
Maximum arc (°)	89.6 (78.3–127.3)	87.3 (80.5–121.1)	119.6 (102.7–127.9)	88.4 (70.0–128.0)	0.462

*FCT* Fibrous cap thickness

Values are n (%) or median (25th, 75th percentiles)

*p* < 0.05 for ^a^proximal versus middle versus distal; ^b^proximal versus middle; ^c^middle versus distal; ^d^proximal versus distal

#### Plaque level

A total of 148 analyzable plaques (1.95 ± 1.07 per patient) were found in the RAP group, including 72 (48.6%) fibrous, 50 (33.8%) lipid, and 26 (17.6%) calcified plaques. RAP tended to cluster in the distal (42.6%) and proximal (37.2%) segments; particularly, calcified plaques were located more distally. Median plaque length was 4.9 (2.3–8.3) mm and 8.8% showed diffuse lesions. Fibrous caps had a median thickness of 220 (140–330) μm. Of the 25 lipid-rich plaques, 24 were thick-cap fibroatheroma and one was thin-cap fibroatheroma. The quantitative analysis of the plaques was comparable among the three segments except for the maximal depth of calcified plaque in the distal segment, which was slightly higher (*p* = 0.049) (Table 2, Additional file 1: Table S1 and Figure S5).

### Factors related to the presence of RAP

Using a stepwise multivariate logistic regression model, we identified three significant predictors associated with the development of RAP. Expressed as odds ratio (OR) with a 95% confidence interval (CI), these were age (OR: 1.10; 95% CI 1.06–1.14; *p* < 0.001), diabetes (OR: 2.12; 95% CI 1.04–4.33; *p* = 0.038), and smoking history (OR: 3.44; 95% CI 1.40‒8.45; *p* = 0.007) (Table 3).

**Table 3:** Univariate and multivariate logistic regression for RAP presence

Variables	Univariate			Multivariate		
	*P* value	OR	95% CI	*P* value	OR	95% CI
Age	< 0.001	1.089	1.058–1.120	< 0.001	1.101	1.064–1.139
Males	0.131	0.606	0.317–1.160	0.484	0.707	0.267–1.870
Smoker	0.485	1.229	0.688–2.195	0.007	3.443	1.404–8.445
Body mass index	0.231	0.949	0.872–1.034	0.554	0.969	0.872–1.076
Diabetes mellitus	0.029	1.944	1.069–3.535	0.038	2.124	1.043–4.329
Hypertension	0.055	1.723	0.989–3.003	0.296	1.443	0.725–2.873
Hyperlipidemia	0.227	1.504	0.775–2.916	0.105	1.955	0.870–4.394
Previous stroke	0.010	3.965	1.384–11.354	0.234	2.167	0.607–7.733
Renal insufficiency	0.014	4.676	1.362–16.053	0.525	1.630	0.362–7.347
Peripheral vascular disease	0.725	1.299	0.302–5.581	0.574	0.614	0.112–3.359

### RAP and the severity of coronary atherosclerosis

**Fig. 2:**
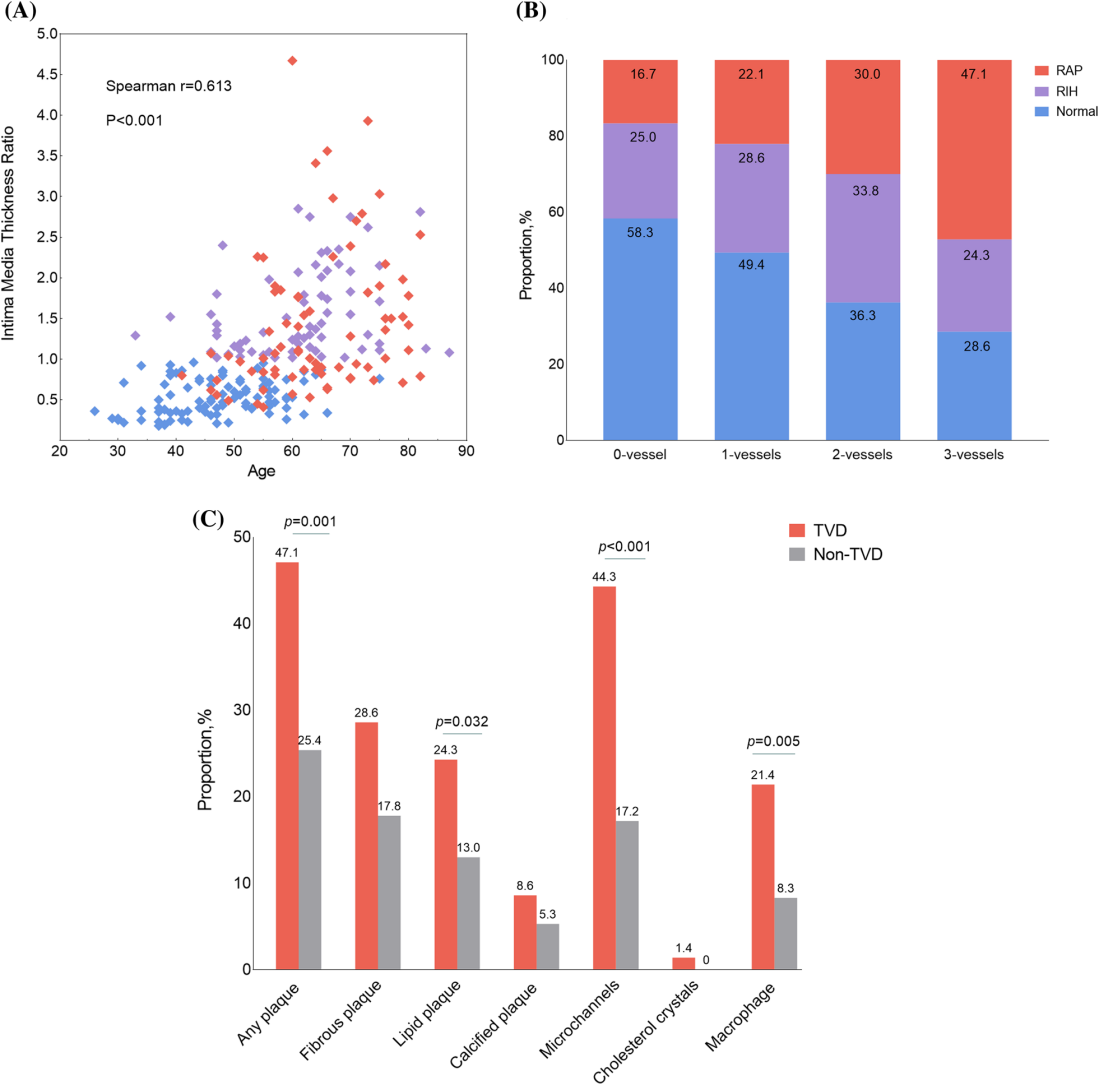
Intimal hyperplasia and atherosclerosis of RA and its clinical correlation. **A** Scatterplots showing a significant positive correlation between the intima media ratio of non-RAP regions and age, indicated by a filled square for each patient in normal (blue), RIH (purple) or RAP (red) groups. **B** The Number of diseased coronary arteries is compared with RA groups. **C** The RA atherosclerotic lesion characters in patients with and without TVD. RA, radial artery; RAP, radial artery plaque; RIH, radial artery intimal hyperplasia; TVD, triple vessel disease

**Fig. 3:**
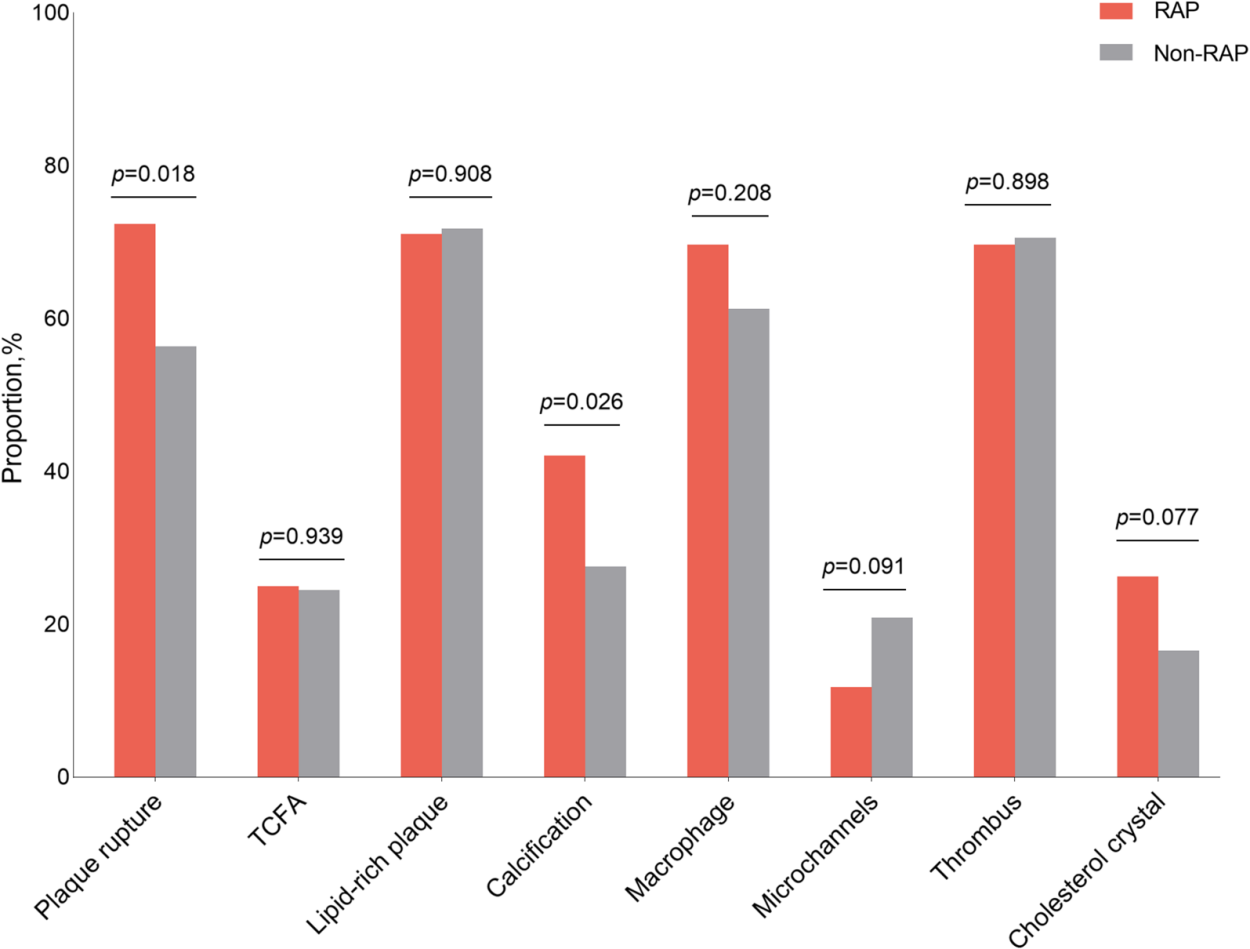
OCT characteristics of coronary artery culprit lesion in patients with or without RAP. Values are n (%). OCT, optical coherence tomography; RAP radial artery plaque; TCFA, thin cap fibroatheroma

A significant correlation between RAP present and the number of coronary arteries with 50% stenosis on angiography was observed (*p* = 0.024), as shown in Fig. 2B. Furthermore, compared with patients with non-TVD, patients with TVD had a higher prevalence of lipid plaques (24.3% vs. 13.0%, *p* = 0.032) and more extensive lipid involvement [7.10 (4.10–11.30) vs. 4.15 (2.53–6.60) mm, *p* = 0.049]. Microvessels were observed in 44.3% of patients with TVD and 17.2% of non-TVD patients (*p* < 0.001) (Fig. 2C and Additional file 1: Table S2). Coronary culprit lesion characteristics evaluated by OCT are listed in Fig. 3 and Additional file 1: Table S5. Compared with non-RAP patients, Patients with RAP have more frequently plaque rupture (72.4% vs. 56.4%, *p* = 0.018) and more frequently calcification (42.1% vs. 27.6%, *p* = 0.026) at culprit lesion.

### Quantitative comparison among groups

Quantitative findings relating to structure are shown in Table 4. The MIT, IMR, and IEI of all three segments were higher in the RAP and RIH groups than the normal group (*p* < 0.001), but comparable between the RAP and RIH groups. Patients in the RAP and RIH groups had a larger intimal area (*p* < 0.001), but lumen area, diameter, and medial area were similar among the three groups. Additionally, the RAP group presented with more severe luminal narrowing, followed by the RIH and normal groups (*p* = 0.002). Details are shown in Additional file 1: Table S3. Multivariate linear regression analysis confirmed that age and gender were independent predictors of IMR (*p* < 0.001 and *p* = 0.016, respectively, Additional file 1: Table S4). Scatterplot depicted an inverse relationship between age and IMR (Spearman’s r: 0.613, *p* < 0.001, Fig. 2A).

**Table 4:** Optical coherence tomography analysis of structure parameters

	Normal group (n = 94)	RIH group (n = 69)	RAP group (n = 76)	*P*
LA, mm^2^	6.60 (4.92–8.34)	6.58 (4.53–8.16)	5.71 (4.63–7.46)	0.349
MD, mm	2.87 (2.51–3.22)	2.89 (2.40–3.21)	2.68 (2.42–3.05)	0.313
MIT, mm	0.08 (0.05–0.10)	0.16 (0.13–0.19)^a^	0.17 (0.12–0.20)^b^	< 0.001
IMR	0.50 (0.35–0.72)	1.33 (1.11–1.90)^a^	1.10 (0.81–1.83)^b^	< 0.001
IEI	1.81 (1.50–2.31)	3.08 (2.75–3.51)^a^	3.05 (2.75–3.78)^b^	< 0.001
ITI	0.41 (0.35–0.50)	0.51 (0.44–0.62)^a^	0.52 (0.42–0.67)^b^	< 0.001
%LN	25.94 (23.54–30.40)	26.83 (24.52–29.75)	29.19 (26.58–32.24)^b,c^	0.002
Intima area, mm^2^	0.62 (0.49–0.84)	0.80 (0.58–0.96)^a^	0.82 (0.66–1.03)^a,b^	< 0.001
Media area, mm^2^	1.58 (1.35–1.93)	1.58 (1.16–2.03)	1.54 (1.31–1.81)	0.922

*LA* lumen area, *MD* mean diameter, *MIT* maximal intimal thickness, *IMR* intima/media thickness ratio, *IEI* Intimal eccentricity index, *ITI* intimal area/medial area, *%LN* luminal narrowing index, others as in Table 1

Values are n (%), or median (25th, 75th percentiles)

*p* < 0.05 for ^a^normal versus RIH; ^b^normal versus RAP; ^c^RIH versus RAP

## Discussion

To our knowledge, this study represents the first systematic effort to use OCT to characterize the plaque morphology of the RA in ACS patients. The major findings are as followings: (1) RAP was present in 31.8% of ACS patients, sharing similar characteristics with coronary plaque, with plaque predominantly mild and focal in nature but not uncommon and combined with obvious intimal hyperplasia in non-RAP segments; (2) aging, diabetes, and smoking are independent risk factors for the presence of RAP.

### In vivo detection of RA atherosclerotic lesions

Previous studies have demonstrated that atherosclerosis is a systemic process involving most artery types, including RA [14, 18]. However, the limited tissue sampling from autopsies and the discarded ends of grafts probably underestimates RA disease [6, 14, 19, 20]. Intravascular ultrasound further revealed a higher percentage of atheroma volume of the RA proximal portion in CAD patients than in healthy controls [10], but was inadequate for indicating subtle structural characteristics. High-resolution OCT is a feasible modality improving insight into the RA in vivo [13, 21, 22]. In our study, OCT-defined RAP was found in 31.8% of ACS patients, which was higher than previous studies in which it ranged from 5.3 to 31.5% [9, 14, 19].This difference might be attributable to differences in study population, evaluation methods, and indexes. Additionally, in our study the angioplasty wire was removed before OCT imaging to avoid guidewire artifacts affecting image interpretation.

We observed that the RA atherosclerotic lesions shared common features with coronary-type fibrous, lipid, and calcified plaque, yet were free of plaque rupture or obstructive lesion, behaving indolently and quiescently relative to coronary artery atherosclerosis. Previous studies using OCT proved that patients with plaque rupture share a common phenotype of more diffuse atherosclerotic process and have a worse prognosis [23, 24]. Our study indicated that patients with RAP exhibited more frequently plaque rupture and calcification in culprit lesion. Thus, the prognosis value of RAP in ACS patients deserve further study.

The incidence of fibrous, lipid, and calcified plaque in our study was 48.6%, 33.8%, and 17.6%, respectively. Fibrous plaque was most common and was distributed evenly among the three segments. Most of the lipid composition was located deep within the plaque, underneath a poorly formed layer. However, vascular response assays indicated that RA with mild or severe atherosclerotic lesions undergoes altered vasoconstrictive response and electrophysiological properties, both with reduced compliance [25] and higher resting membrane potential [26] compared with normal vessels. Brown et al. [27] reported that detection of intimal lipids by OCT in the RA could predict the extent of postoperative spasm of grafts. Although calcium deposition was more frequently associated with atherosclerosis and regarded as an excellent marker for plaque burden, the calcified plaques in RA are infrequent and comparable among patients with different severity of coronary atherosclerosis. Interestingly, similar patterns exist beyond coronary arteries such as calcified nodules protruding into the RA lumen [28]. The mechanism of calcium deposition in the RA and cardiovascular physiology remains unclear.

Microvessels can also be visualized in the RA, confined to the adventitia, and occur normally when the vessel wall thickens, but are also stimulated by inflammation or atherosclerosis [29]. The accelerated intimal hyperplasia of the RA conduit is secondary to ischemia, reportedly caused by deprivation of its vasa vasorum and lymphatic drainage [30].

### Underlying intimal hyperplasia in RA

The discontinuities of internal elastic lamina in RA were related with the progressive intimal hyperplasia and atherosclerosis [6]. Previous studies showed that excessive intimal thickening is the first step in coronary pre-atherosclerosis, preceding lipid deposition [31–33]. Indetectable but important, therefore, the IMR has been widely used for evaluating the severity of intimal hyperplasia [13, 14, 34, 35].

Notably, we identified a trend manifested as obvious intimal hyperplasia combined with mature plaque involving the RA in patients presenting with overt coronary disease. However, no differences in the media layer were demonstrated among our three groups, a similar observation to that made using ultrabiomicroscopy in a comparison between patients with and without CAD [4]. Xu and associates reported that RA intimal thickness measured by ultrasound biomicroscopy could detect CAD independently similarly to that of carotid intima-media thickness [3]. Thus we support that investigating the intima separately from the media layer rather than investigating the intima-media complex may shed more light on the early stages of atherosclerosis [3]. The intimal hyperplasia of the eccentric pattern has been seen in coronary, carotid, cerebral, and renal arteries, some of which coincides with atherosclerosis susceptible regions [36]. We also found such crescent-shaped intimal hyperplasia in RA.

### Clinical characteristics of RA atherosclerosis

In general, the severity of arteriosclerosis increases with advancing age in both coronary and peripheral arteries and the RA is no exception [37]. In patients in the fifth decade of life, RA atherosclerosis has progressed beyond the early stages, yet no rational guidelines have been recommended on the applicable age for using the RA conduit until now. Moreover, RA provides the best vascular access for hemodialysis, yet pre-existing intimal hyperplasia in patients with diabetes or old age may explain the high incidence of radiocephalic arteriovenous fistula failure [38]. Care should be taken when using the RA in the elderly. In accordance with previous findings [6, 10, 39], smoking and diabetes were found in our study to act as a trigger for accelerating RAP formation. Research has stated that in smokers, the RA is less responsive to acetylcholine and has enhanced reactive oxygen species compared with the internal mammary artery [40]. Subtle intimal defects caused by atherosclerosis provide potential risk of spasm and injury in revascularization, and may even be a mechanism for late graft failure [41–43]. Our results indicated that the middle segment of the RA is a relatively better selection for high-risk coronary artery bypass graft (CABG) patients.

### Limitations

The present study is limited by its observational nature, so the possibility of bias exists. The study did not provide series observation of the whole RA and was not controlled with healthy patients. Atherosclerosis is a multifactorial disease, and genetic, ethnic, and lifestyle issues cannot be ignored. In addition, the patients in this study with dyslipidemia, hypertension, and diabetes were being treated; that these treatments may have mitigated any potential effects on intimal hyperplasia and plaque formation cannot be excluded. Hence, these findings are insufficient to enable strong inferences to be made about underlying pathophysiology. Although we excluded patients with a history of transradial coronary intervention (TRI) [44], the effect of acute changes from invasive examination is still a limitation. Meanwhile, those excluded patients with repeat coronary revascularization may have worse vasculature independent of TRI-induced atherosclerosis. The application of OCT is undeniably at the preliminary stage of research. Currently, the classification of OCT-determined intimal change without differentiation from its pathological counterpart may confuse the assessment of atherosclerotic lesions.

## Conclusions

This study demonstrates that OCT without guidewire shadow is a promising modality for identifying plaque and early intimal hyperplasia of the RA in vivo. RAP is a not uncommon finding in patients with ACS, accounting for 31.8% of cases in the present study. In addition, RA atherosclerotic lesions share some common characteristics with coronary atherosclerosis and relate to the severity of ACS. Care should be taken in patients with advanced age, diabetes, and smoking history.

## Supplementary Information

**Additional file 1. Table S1.** OCT findings of atherosclerosis characters. **Table S2.** OCT findings in patients with and without TVD. **Table S3.** OCT structure parameters of RA in segment level. **Table S4.** Multivariate regression analysis of IMR. **Table S5.** OCT characteristics of coronary culprit lesion in patients with or without RAP. **Figure S1.** Study flow. **Figure S2.** Schematic representation of OCT observation of the RA. **Figure S3.** Morphometric measurements of RA by OCT in segment level. **Figure S4.** OCT imaging and measurements of crescent-shaped intimal hyperplasia. **Figure S5.** The distribution of RA plaques.

## Data Availability

The datasets used and analyzed during the current study are available from the corresponding author on reasonable request.

## Abbreviations

ACS: Acute coronary syndrome
CAD: Coronary artery disease
IMR: Intima media thickness ratio
OCT: Optical coherence tomography
PCI: Percutaneous coronary intervention
RA: Radial artery
RAP: Radial artery plaque
RIH: Radial artery intimal hyperplasia
