# Supplementary material for: Assessment of radial artery atherosclerosis in acute coronary syndrome patients: an in vivo study using optical coherence tomography
Source: BMC Cardiovasc Disord. 2022 Mar 21;22:120. doi: 10.1186/s12872-022-02561-5 (PMC8939080; doi:10.1186/s12872-022-02561-5)
Supplement: Supplementary file 1 — Additional file 1. Table S1. OCT findings of atherosclerosis characters. Table S2. OCT findings in patients with and without TVD. Table S3. OCT structure parameters of RA in segment level. Table S4. Multivariate regression analysis of IMR. Table S5. OCT characteristics of coronary culprit lesion in patients with or without RAP. Figure S1. Study flow. Figure S2. Schematic representation of OCT observation of the RA. Figure S3. Morphometric measurements of RA by OCT in segment level. Figure S4. OCT imaging and measurements of crescent-shaped intimal hyperplasia. Figure S5. The distribution of RA plaques. [file 12872_2022_2561_MOESM1_ESM.docx]

**Supplemental Materials**

**Supplemental methods**

***OCT analysis***

Detailed optical coherence tomographic (OCT) assessment of radial artery(RA) included luminal diameter, lumen area (LA), internal elastic lamina area (IEL) and external elastic lamina area (EEL). Then intimal area (IEL area minus luminal area) and medial area (EEL area minus IEL area) were calculated, respectively. Intima thickness was defined as the distance between the lumen and the IEL, the corresponding media thickness defined as the distance between the IEL and EEL was also recorded. The structural parameters were obtained from defined sites at 90 and 180 cross-sectional frames in proximal, middle, distal segment respectively. Then at the cross-sectional frame with the maximum intimal thickness (MIT), width of the intima at maximal and minimal intimal thickness and width of the media at maximal intimal thickness.

The severity of intimal hyperplasia in the RA included (i) percentage of luminal narrowing=(intimal area+ media area) /IEL area ×100, (ii) intimal thickness index (ITI)=intimal area/medial area, (iii) intima media thickness ratio (IMR)=MIT/width of media at maximal intima thickness, and (iv) intimal eccentricity index (IEI)=MIT/minimum intimal thickness [1-3].

Lipid-rich plaque was defined as maximal lipid arc greater than 90°. Thin-cap fibroatheroma (TCFA) was defined as lipid arc≥1 quadrant with fibrous cap <65μm. Spotty calcifications was defined as the presence of lesions with maximal arc <90° and length <4 mm. Large calcifications were defined as lesions with maximal arc ≥90° or length ≥ 4 mm [4, 5]. OCT analysis of coronary artery was conducted according to culprit lesion morphology. Culprit lesion was identified by means of angiography, electrocardiographic ST-segment alterations, and/or regional wall motion abnormalities on echocardiographic assessment. Plaque rupture was defined as the presence of fibrous cap discontinuity with or without cavity formation. Intact fibrous cap was defined as a plaque where the fibrous cap of the culprit lesion was intact[6] [7].

**Supplemental tables and figures**

**Table S1.** OCT Findings of atherosclerosis characters.

**Table S2.** OCT findings in patients with and without TVD.

**Table S3.** OCT structure parameters of RA in segment level.

**Table S4.** Multivariate regression analysis of IMR.

**Figure S1.** Study flow.

**Figure S2.** Schematic representation of OCT observation of the RA.

**Figure S3.** Morphometric measurements of RA by OCT in segment level.

**Figure S4.** OCT imaging and measurements of crescent-shaped intimal hyperplasia.

**Figure S5.** The distribution of RA plaques.

**Table S1. OCT Findings of atherosclerosis characters**

| **Patient-level** |  |
| --- | --- |
| Plaque present | n=76 |
| Total lesion length, mm | 7.05(3.23-13.08) |
| Single/Multiple plaque type | 51(67.1)/25 (32.9) |
| Fibrous plaque | 50/239(20.9) |
| Lipid plaque | 39/239(16.3) |
| Calcified plaque | 15/239(6.3) |
| Lesion Segments |  |
| Single / Multiple involvement | 39(51.3)/37(48.7) |
| Proximal | 47(19.7) |
| Middle | 28(11.7) |
| Distal | 50(20.9) |
| Microstructure present |  |
| Cholesterol crystals | 1(0.4) |
| Microchannels | 60(25.1) |
| Macrophage accumulation | 29(12.1) |
| **Plaque-level** |  |
| The number of plaques | n=148 |
| Plaque length, mm | 4.85 (2.33-8.30) |
| Diffuse lesion(>20mm) | 13(8.8) |
| Plaque type |  |
| Fibrous plaque | 72(48.6) |
| Length, mm | 3.40(1.60-7.70) |
| Lipid plaque | 50 (33.8) |
| Length, mm | 4.90 (3.08-6.92) |
| Fibrous cap thickness, μm | 220(140-330) |
| Maximum lipid arc, ° | 89.6(78.3-127.3) |
| Lipid-rich | 24 |
| Thin-cap fibroatheroma | 1 |
| Calcified plaque | 26(17.6) |
| Spotty/ Large | 5/21 |
| Length, mm | 8.1(4.6-20.8) |
| Maximal depth, mm | 0.21(0.16-0.33) |
| Maximum calcium arc, ° | 62.8(43.2-101.7) |

Values are mean±SD, median (25th, 75th percentiles) or n (%).

**Table S2. OCT findings in patients with and without TVD**

| **Variables** | **Non-TVD**  **(n=169)** | **TVD**  **(n=70)** | ***p*** |
| --- | --- | --- | --- |
| Any plaque | 43(25.4) | 33(47.1) | **0.001** |
| Fibrous | 30(17.8) | 20(28.6) | 0.061 |
| Lipid | 22 (13.0) | 17 (24.3) | **0.032** |
| Calcified | 9(5.3) | 6(8.6) | 0.517 |
| Microchannels | 29(17.2) | 31(44.3) | **<0.001** |
| Cholesterol crystals | 0 | 1(1.4) | … |
| Macrophage accumulation | 14(8.3) | 15(21.4) | **0.005** |
| Total lesion length, mm | 6.1(3.1-13.5) | 7.4(3.6-12.7) | 0.615 |
| Fibrous, mm | 5.4(2.6-10.3) | 3.2(1.9-7.1) | 0.205 |
| Lipid, mm | 4.2(2.5-6.6) | 7.1(4.1-11.3) | **0.049** |
| Calcified, mm | 8.3(3.4-36.0) | 15.3(8.8-63.6) | 0.181 |
| Plaque involvement |  |  |  |
| Single segment | 23(13.6) | 16(22.9) | 0.078 |
| Multiple segments | 20(11.8) | 17(24.3) | **0.015** |

Values are mean±SD, median (25th, 75th percentiles) or n (%).

TVD, triple vessel disease

**Table S3** **Morphometric measurements of RA by OCT in segment level**

|  | **Normal group**  **(n=94)** | **RIH group**  **(n=69)** | **RAP group**  **(n=76)** | ***p*** |
| --- | --- | --- | --- | --- |
| LA (mm^2^) |  |  |  |  |
| Total | 6.60(4.92-8.34) | 6.58(4.53-8.16) | 5.71(4.63-7.46) | 0.349 |
| Pro | 6.11(4.39-8.70) | 6.29(4.23-8.41) | 5.46(4.08-7.45) | 0.222 |
| Mid | 7.06(5.18-8.95) | 6.78(4.72-8.47) | 5.67(4.80-8.70) | 0.340 |
| Dis | 5.91(4.38-8.15) | 5.73(4.70-8.29) | 5.44(4.54-7.00) | 0.521 |
| MD (mm) |  |  |  |  |
| Total | 2.87(2.51-3.22) | 2.89(2.40-3.21) | 2.68(2.42-3.05) | 0.313 |
| Pro | 2.79(2.39-3.31) | 2.81(2.30-3.27) | 2.64(2.27-3.07) | 0.177 |
| Mid | 2.94(2.60-3.41) | 2.94(2.45-3.28) | 2.71(2.47-3.29) | 0.287 |
| Dis | 2.72(2.37-3.21) | 2.70(2.45-3.29) | 2.61(2.40-3.00) | 0.485 |
| MIT (mm) |  |  |  |  |
| Total | 0.08(0.05-0.10) | 0.16(0.13-0.19) ^a^ | 0.17(0.12-0.20) ^b^ | **<0.001** |
| Pro | 0.07(0.05-0.10) | 0.15(0.10-0.20) ^a^ | 0.15(0.09-0.21) ^b^ | **<0.001** |
| Mid | 0.06(0.04-0.09) | 0.16(0.11-0.20) ^a^ | 0.15(0.10-0.22) ^b^ | **<0.001** |
| Dis | 0.08(0.06-0.12) | 0.17(0.14-0.21) ^a^ | 0.19(0.13-0.24) ^b^ | **<0.001** |
| IMR |  |  |  |  |
| Total | 0.50(0.35-0.72) | 1.33(1.11-1.90) ^a^ | 1.10(0.81-1.83) ^b^ | **<0.001** |
| Pro | 0.33(0.24-0.54) | 0.94(0.60-1.60) ^a^ | 0.85(0.45-1.37) ^b^ | **<0.001** |
| Mid | 0.38(0.28-0.58) | 1.29(0.93-2.11) ^a^ | 1.08(0.70-1.70) ^b^ | **<0.001** |
| Dis | 0.60(0.38-0.88) | 1.62(1.20-2.24) ^a^ | 1.30(0.89-1.98) ^b^ | **<0.001** |
| IEI |  |  |  |  |
| Total | 1.81(1.50-2.31) | 3.08(2.75-3.51) ^a^ | 3.05(2.75-3.78) ^b^ | **<0.001** |
| Pro | 1.67(1.38-2.18) | 3.00(2.19-3.88) ^a^ | 3.00(2.00-3.97) ^b^ | **<0.001** |
| Mid | 1.63(1.33-2.00) | 3.00(2.20-3.92) ^a^ | 2.75(2.00-3.74) ^b^ | **<0.001** |
| Dis | 2.00(1.67-2.45) | 3.00(2.50-3.93) ^a^ | 3.21(2.33-4.09) ^b^ | **<0.001** |
| ITI |  |  |  |  |
| Total | 0.41(0.35-0.50) | 0.51(0.44-0.62) ^a^ | 0.52(0.42-0.67) ^b^ | **<0.001** |
| Pro | 0.36(0.29-0.48) | 0.44(0.37-0.54) ^a^ | 0.44(0.33-0.54) ^b^ | **0.002** |
| Mid | 0.38(0.33,0.47) | 0.51(0.42-0.64) ^a^ | 0.49(0.41-0.62) ^b^ | **<0.001** |
| Dis | 0.45(0.37-0.56) | 0.57(0.44-0.71) ^a^ | 0.60(0.46-0.73) ^b^ | **<0.001** |
| %LN |  |  |  |  |
| Total | 25.94(23.54-30.40) | 26.83(24.52,29-75) | 29.19(26.58-32.24) ^b^ ^c^ | **0.002** |
| Pro | 26.79(22.40-33.15) | 27.79(24.93-31.12) | 31.38(26.55-36.27) ^b^ | **0.002** |
| Mid | 24.15(19.87-27.93) | 24.62(22.65-28.13) | 26.58(23.72-30.25) ^b^ | **0.010** |
| Dis | 26.42(22.57-30.52) | 26.63(23.84-31.33) | 29.65(25.59-32.67) ^b^ | **0.013** |
| Intimal area(mm^2^) |  |  |  |  |
| Total | 0.62(0.49-0.84) | 0.80(0.58-0.96) ^a^ | 0.82(0.66-1.03) ^b^ | **<0.001** |
| Pro | 0.59(0.45-0.91) | 0.79(0.55-0.97) | 0.76(0.58-1.02) ^b^ | **0.006** |
| Mid | 0.59(0.44-0.74) | 0.73(0.55-0.97) ^a^ | 0.81(0.59-0.97) ^b^ | **<0.001** |
| Dis | 0.66(0.50-0.84) | 0.80(0.61-1.08) ^a^ | 0.89(0.74-1.08) ^b^ | **<0.001** |
| Media area(mm^2^) |  |  |  |  |
| Total | 1.58(1.35-1.93) | 1.58(1.16-2.03) | 1.54(1.31-1.81) | 0.922 |
| Pro | 1.75(1.39-2.14) | 1.69(1.27-2.22) | 1.71(1.43-2.08) | 0.950 |
| Mid | 1.57(1.26-1.83) | 1.55(1.09-1.91) | 1.50(1.28-1.72) | 0.771 |
| Dis | 1.41(1.24-1.76) | 1.56(1.05-1.84) | 1.47(1.26-1.75) | 0.961 |

Values are median (25th, 75th percentiles).

*p* < 0.05 for ^a^ Normal vs. RIH; ^b^ Normal vs. RAP; ^c^ RIH vs. RAP

Total, average value in total; Pro, proximal, 50mm from the ostium; Mid, middle, 100mm from the ostium; Dis, distal,100mm away the ostium; OCT, optical coherence tomography; LA, lumen area; MD, mean diameter; MIT, maximal intimal thickness; IMR, MIT/media thickness measured at the MIT; IEI, Intimal eccentricity index; ITI, intima area/media area; %LN, luminal narrowing index; RIH, radial artery intimal hyperplasia; RAP, radial artery plaque.

**Table S4. Multivariate regression analysis of IMR**

|  | **Univariate** | | **Multivariate** | |
| --- | --- | --- | --- | --- |
|  | β Coeff (95%CI) | p Value | β Coeff (95%CI) | p Value |
| Age (years) | 0.530(0.025, 0.038) | <0.001 | 0.480 (0.022, 0.035) | <0.001 |
| Female | 0.311(0.351, 0.800) | <0.001 | 0.142(0.050,0.474) | 0.016 |
| BMI (kg/m^2^) | 0.014(-0.026, 0.032) | 0.827 | … |  |
| Smoker | -0.200(-0.511, -0.117) | 0.002 | … |  |
| Diabetes mellitus | 0.142(0.026, 0.449) | 0.028 | … |  |
| Hypertension | 0.178(0.078, 0.455) | 0.006 | … |  |
| Hyperlipidemia | -0.052(-0.311, 0.132) | 0.426 | … |  |
| Previous stroke | 0.163(0.108, 0.863) | 0.012 | … |  |
| Renal insufficiency | 0.124(-0.10, 0.858) | 0.055 |  |  |
| Familial history of CAD | -0.008(-0.290, 0.257) | 0.907 | … |  |
| LDL-C (mg/dl) | -0.028(-0.003, 0.002) | 0.672 | … |  |
| Creatinine(μmmol/L) | 0.010(-0.005, 0.006) | 0.882 | … |  |

**Table S5 OCT characteristics of** **coronary culprit lesion in patients with or without RAP**

| Variables | Non-RAP group  (n=163) | RAP group  (n=76) | p Value |
| --- | --- | --- | --- |
| Culprit artery |  |  | 0.125 |
| LAD | 92 (56.4%) | 34 (44.7%) |  |
| RCA | 49 (30.1%) | 33 (43.4%) |  |
| LCX | 22 (13.5%) | 9 (11.8%) |  |
| Culprit lesion |  |  | 0.018 |
| Plaque rupture | 92 (56.4%) | 55 (72.4%) |  |
| Intact fibrous cap | 71 (43.6%) | 21 (27.6%) |  |
| Plaque composition |  |  | 0.908 |
| Lipid-rich plaque | 117 (71.8%) | 54 (71.1%) |  |
| Fibrous plaque | 46 (28.2%) | 22 (28.9%) |  |
| Calcification | 45 (27.6%) | 32 (42.1%) | 0.026 |
| TCFA | 40 (24.5%) | 19 (25.0%) | 0.939 |
| Macrophage | 100 (61.3%) | 53 (69.7%) | 0.208 |
| Microchannels | 34 (20.9%) | 9 (11.8%) | 0.091 |
| Cholesterol crystal | 27 (16.6%) | 20 (26.3%) | 0.077 |
| Thrombus | 115 (70.6%) | 53 (69.7%) | 0.898 |

**Figure S1. Study flow**

**
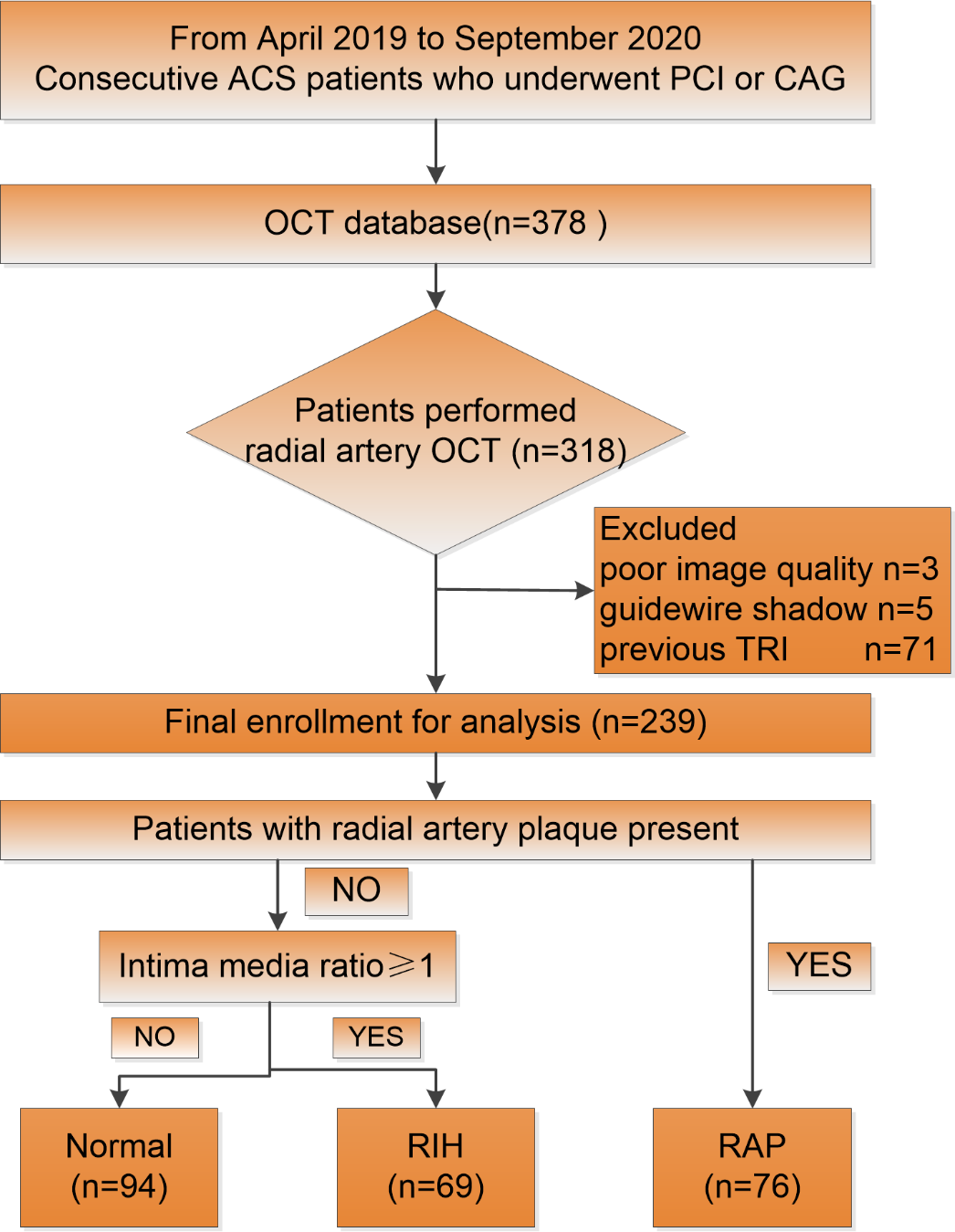
**

ACS, acute coronary syndrome; CAG, coronary angiogram; OCT, optical coherence tomography; PCI, percutaneous coronary intervention; RAP, radial artery plaque; RIH, radial artery intimal hyperplasia; TRI, transradial coronary intervention

**Figure S2.** **Schematic representation of OCT observation of the radial artery.**

**
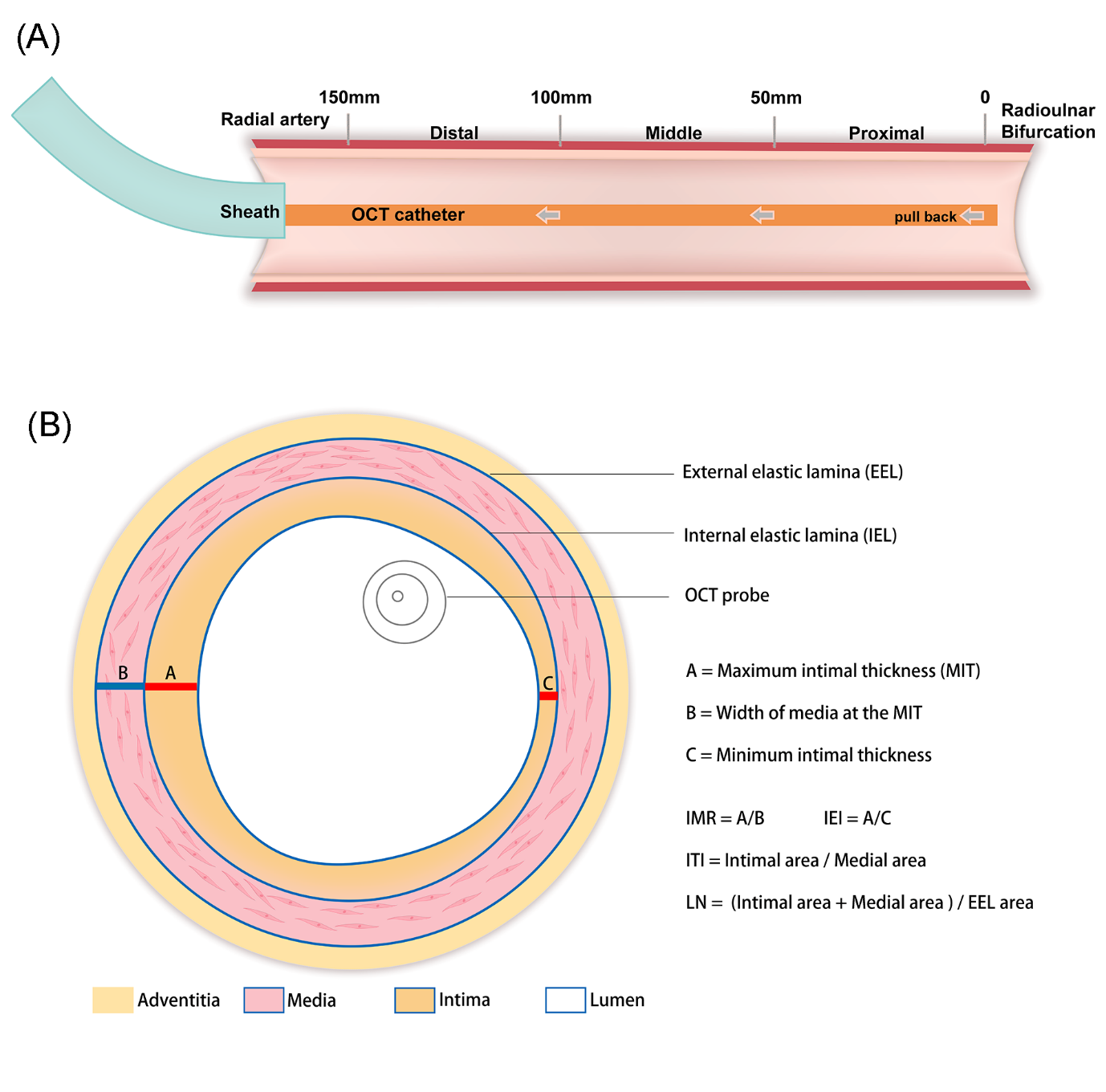
**

(A)Radial artery OCT imaging method. (B) Radial artery structural parameters

**Figure S3.** **Representative case of lipid plaque present in RA by OCT images.**


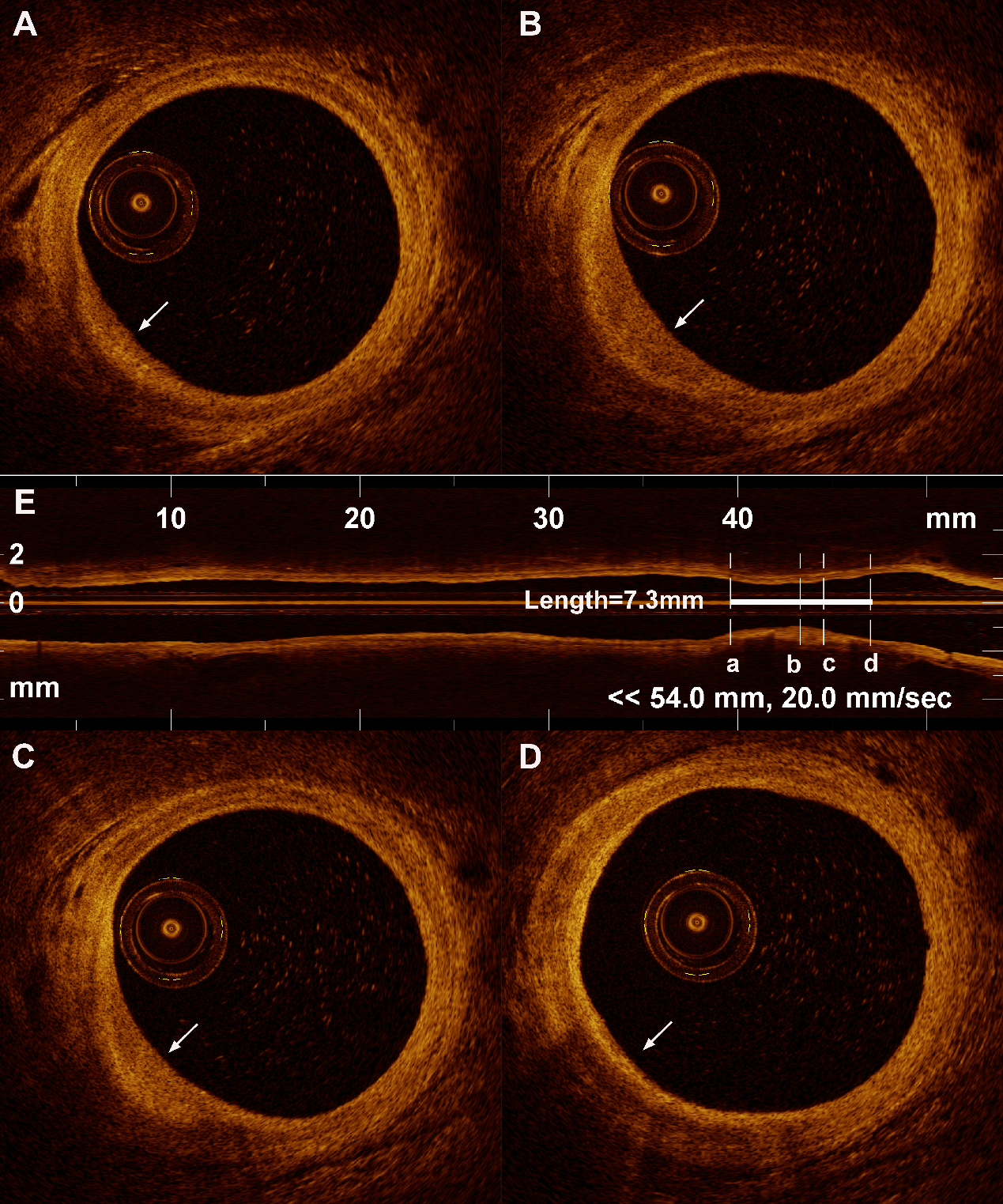


A 71-year-old patient with lipid plaque in distal RA indicated by white arrows. (E) is the longitudinal view of the RA with plaque length 7.3 mm. The position of the selected cross-sectional image indicated by the white line. RA, radial artery.

**Figure S4. Optical coherence tomography imaging and measurements of crescent-shaped intimal hyperplasia**

**
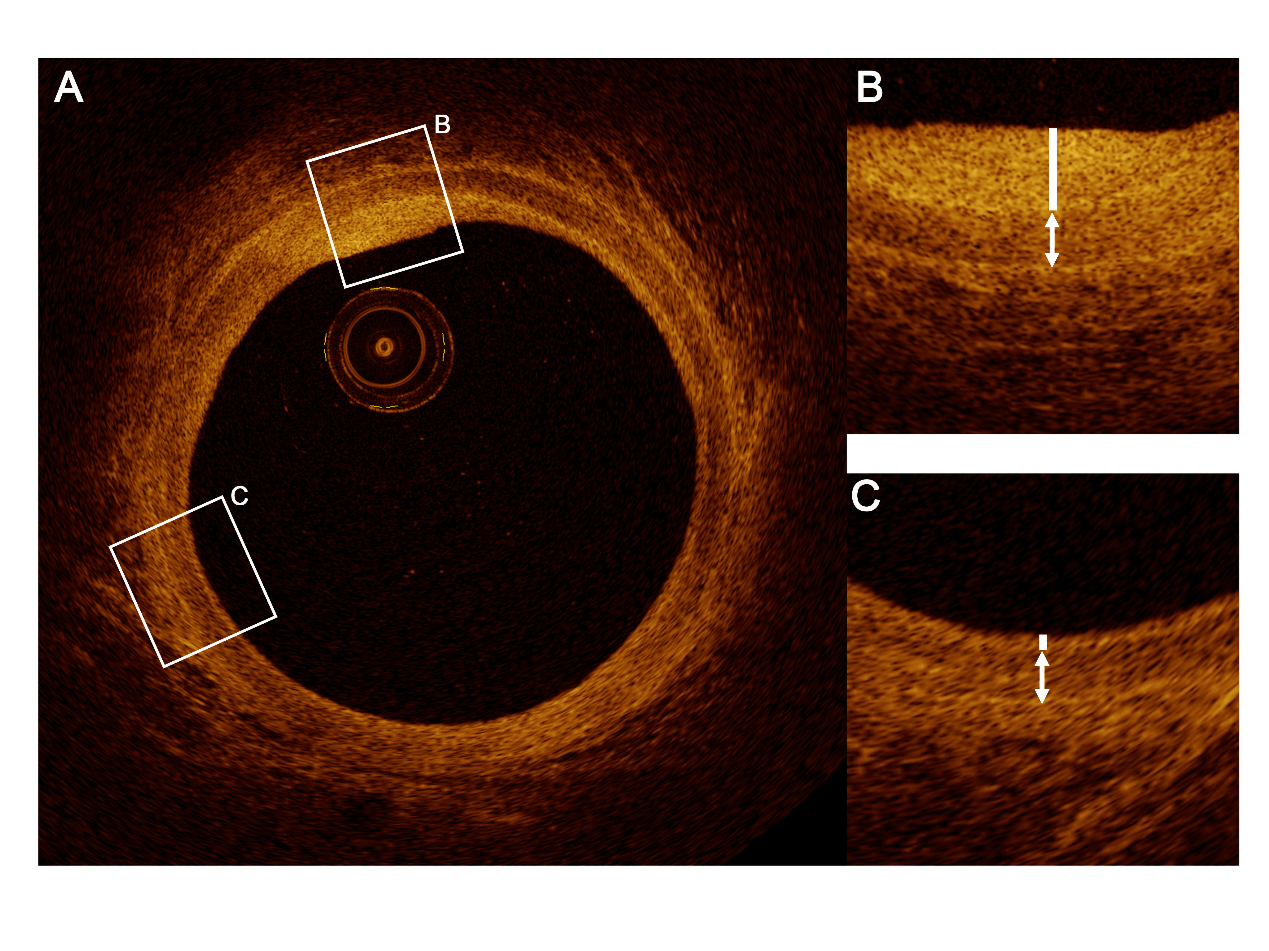
**

(A) shows crescent-shaped intimal hyperplasia with intima media ratio >1 and intimal eccentricity index>4; (B) shows the max intimal thickness=0.33mm (line) and media thickness=0.17mm(arrow); (C) shows min intimal thickness=0.04mm(line).

**Figure S5. The distribution of radial artery plaques.**


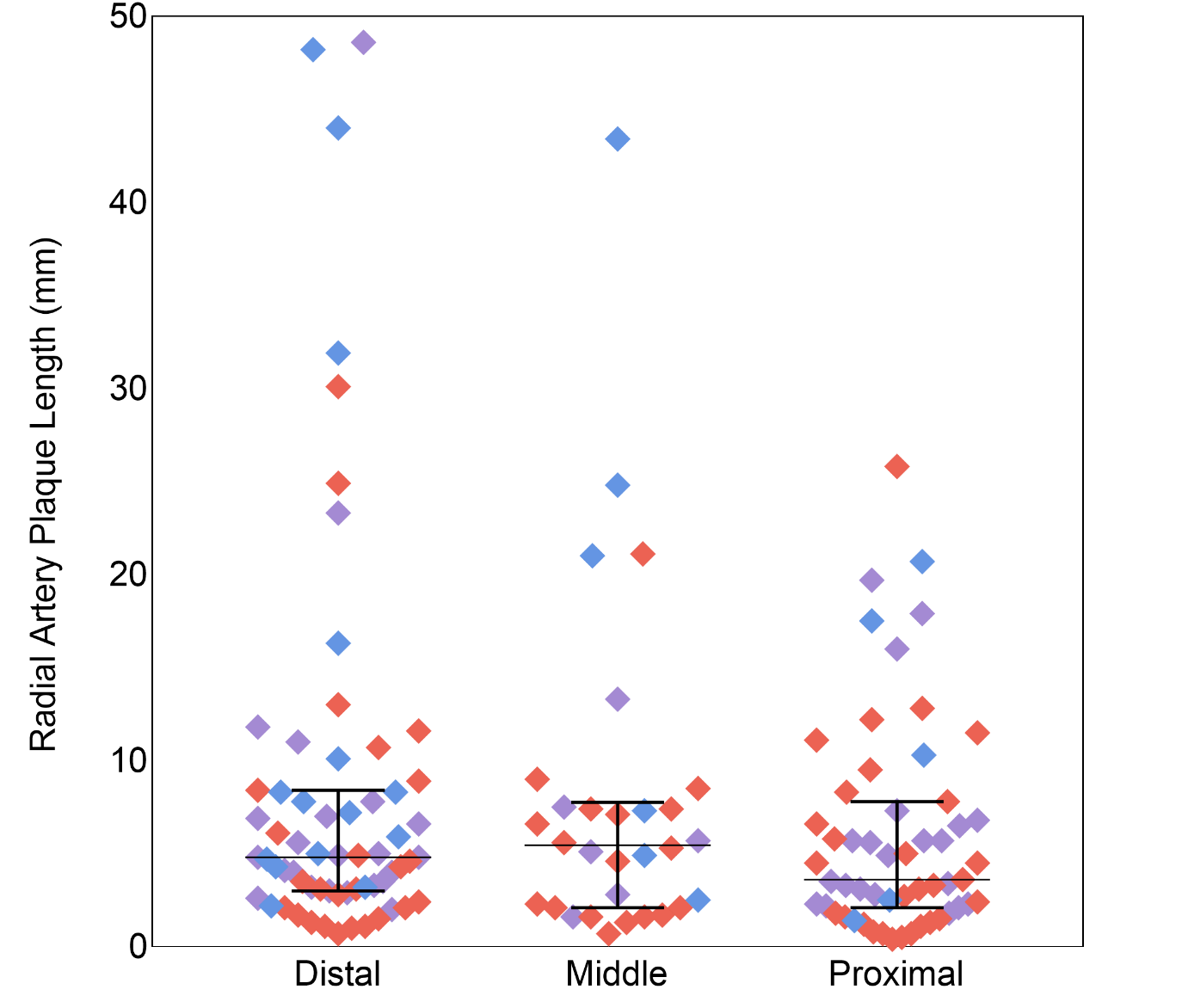


The distribution and length of each radial artery plaque, including fibrous (red), lipid (purple) and calcified (blue) plaques across three segments.

**REFERENCES**

1. Kaufer E, Factor SM, Frame R, Brodman RF: **Pathology of the Radial and Internal Thoracic Arteries Used as Coronary Artery Bypass Grafts**. *The Annals of Thoracic Surgery* 1997, **63**(4):1118-1122.

2. Yonetsu T, Kakuta T, Lee T, Takayama K, Kakita K, Iwamoto T, Kawaguchi N, Takahashi K, Yamamoto G, Iesaka Y *et al*: **Assessment of acute injuries and chronic intimal thickening of the radial artery after transradial coronary intervention by optical coherence tomography**. *Eur Heart J* 2010, **31**(13):1608-1615.

3. Ruengsakulrach P, Sinclair R, Komeda M, Raman J, Gordon I, Buxton B: **Comparative histopathology of radial artery versus internal thoracic artery and risk factors for development of intimal hyperplasia and atherosclerosis**. *Circulation* 1999, **100**(19 Suppl):II139-144.

4. Milzi A, Burgmaier M, Burgmaier K, Hellmich M, Marx N, Reith S: **Type 2 diabetes mellitus is associated with a lower fibrous cap thickness but has no impact on calcification morphology: an intracoronary optical coherence tomography study**. *Cardiovascular diabetology* 2017, **16**(1):152.

5. Ong DS, Lee JS, Soeda T, Higuma T, Minami Y, Wang Z, Lee H, Yokoyama H, Yokota T, Okumura K *et al*: **Coronary Calcification and Plaque Vulnerability: An Optical Coherence Tomographic Study**. *Circ Cardiovasc Imaging* 2016, **9**(1).

6. Niccoli G, Montone RA, Di Vito L, Gramegna M, Refaat H, Scalone G, Leone AM, Trani C, Burzotta F, Porto I *et al*: **Plaque rupture and intact fibrous cap assessed by optical coherence tomography portend different outcomes in patients with acute coronary syndrome**. *Eur Heart J* 2015, **36**(22):1377-1384.

7. Hoshino M, Yonetsu T, Usui E, Kanaji Y, Ohya H, Sumino Y, Yamaguchi M, Hada M, Hamaya R, Kanno Y *et al*: **Clinical Significance of the Presence or Absence of Lipid-Rich Plaque Underneath Intact Fibrous Cap Plaque in Acute Coronary Syndrome**. *J Am Heart Assoc* 2019, **8**(9):e011820.
